# Adherence to antihypertensive treatment in patients at a tertiary hospital in Lima, Peru

**DOI:** 10.17843/rpmesp.2026.431.15462

**Published:** 2026-03-27

**Authors:** Angy Aguilar-Lizarme, José Amado-Tineo

**Affiliations:** 1 Facultad de Medicina, Universidad Nacional Mayor de San Marcos, Lima, Peru.; 2 Hospital Nacional Edgardo Rebagliati Martins, Lima, Peru.

**Keywords:** Patient Compliance, Hypertension, Antihypertensive Agents

## Abstract

In Peru, there are few reports on pharmacological adherence in patients with arterial hypertension. In this context, the present study aimed to determine adherence to antihypertensive treatment in patients treated at a tertiary-level social security hospital in Lima. A cross-sectional study was conducted in 250 hospitalized patients, over 18 years of age, with a diagnosis of primary arterial hypertension on pharmacological treatment. Adherence was evaluated using the Morisky questionnaire (MMAS-8), and variables such as prescribed medication, age, sex, marital status, and educational level were collected. 56.4% of the participants were male and 86.4% were over 60 years old. It was observed that 42% presented low pharmacological adherence and 58% medium adherence, with no cases of high adherence recorded. Monotherapy was the most frequent regimen (48%), with losartan, nifedipine, and enalapril being the most prescribed drugs. Finally, significant differences were identified between adherence to antihypertensive treatment and educational level and the number of drugs.

## INTRODUCTION

Arterial hypertension (HTN) is the most common cardiovascular pathological condition among adults, affecting more than a quarter of the world's population, which is equivalent to more than one billion people ^1^. It is classified into essential or primary HTN, which represents approximately 90-95% of cases, and secondary hypertension (5-10%), associated with renal and endocrine diseases, medication use, among other causes. Adherence to antihypertensive treatment constitutes a key determinant of HTN control. Various factors, such as the complexity of the therapeutic regimen, polypharmacy, and sociodemographic variables, can influence therapeutic compliance ^2^^,^^3^. 

Globally, it is estimated that 43% of hypertensive patients present low adherence to treatment, with higher figures in low- and middle-income countries ^4^. As a result, effective blood pressure control is limited, as only 13.8% of cases were controlled in 2020 ^5^. Evidence has shown that therapeutic non-compliance is directly associated with poorer blood pressure control, greater progression of target organ damage, and a significant increase in cardiovascular risk ^6^.Various international studies have identified factors associated with adherence, such as age, educational level, presence of comorbidities, and treatment complexity ^6^.

In Latvia, for example, a non-compliance rate of 46.2% was reported, with higher adherence among older adults ^7^. In Saudi Arabia, only 42.2% of patients adequately followed the medication regimen, influenced by coexisting diseases and the use of multiple drugs ^8^. In Brazil, 38% of patients without secondary education presented better adherence compared to 12.5% of those who finished secondary school ^9^.

In Peru, there are significant challenges to guaranteeing HTN control due to limitations in cardiovascular risk monitoring and the adequate distribution of medications ^10^. It has been shown that adherence rates to treatment range between 30% and 41% in adult patients treated at primary and secondary level health facilities, which negatively impacts health outcomes and healthcare system costs ^11^. In this context, the study seeks to determine the level of pharmacological adherence to antihypertensive treatment and identify differences according to patient characteristics in a referral hospital.

**KEY MESSAGES**

Motivation for the study. Adherence to antihypertensive treatment is an aspect that has been poorly evaluated in our setting, despite being a key component for the adequate control of arterial hypertension.

Main findings. 58% of patients hospitalized in a tertiary-level facility presented medium adherence, while 42% showed low adherence. Significant differences were identified in adherence according to educational level and the number of drugs consumed.

Public health implications. The identification of differences in adherence to antihypertensive treatment allows for the recognition of potential intervention points and guides the design of strategies to optimize the control of arterial hypertension.

## THE STUDY

### Design and population

An observational cross-sectional study was conducted. The population consisted of patients on pharmacological antihypertensive treatment hospitalized in the internal medicine services of the Hospital Nacional Edgardo Rebagliati Martins, between February and March 2025. This tertiary-level facility has all medical specialties, has approximately 1,600 hospital beds, and six internal medicine services, each with an average of 40 beds.

### Selection, sample, and sampling

The sample size was estimated at 227 participants using the Epi Info version 7.2.5 program, considering a population of unknown size, a margin of error of 5%, a confidence level of 95%, and a design effect of 1. Patients over 18 years of age, with a diagnosis of primary arterial hypertension and on pharmacological antihypertensive treatment for more than three months, who provided informed consent, were consecutively included. Those with evidence of secondary hypertension, hemodynamic instability, incomplete data, or neurological or physical limitations that prevented responding to the survey were excluded.

### Variables

Through the review of the hospital medical records, sociodemographic characteristics were identified: age (recorded years), sex (male and female), educational level (primary, secondary, and higher), marital status (with or without a partner), and clinical variables (comorbidity, reason for admission, and duration of HTN) of the participants. Adherence to pharmacological antihypertensive treatment prior to hospitalization was evaluated using the MMAS-8 (8-item Morisky Medication Adherence Scale) questionnaire ^12^, and the type and number of drugs used were recorded. The data were collected by a medical graduate through a direct interview with the patient, not accepting responses from family members or companions. The MMAS-8 consists of seven items with a dichotomous response (yes = 0 points / no = 1 point), while the eighth item presents a five-point Likert-type response scale. The sum of the score classifies into: low adherence (less than 6), medium adherence (6-7), and high adherence (8 or more) (supplementary material).

### Statistical analysis

The data were entered into Microsoft Excel 2017 and subsequently analyzed with the SPSS version 25 statistical program. Variables were categorized and described using absolute frequencies and percentages. To evaluate differences between categorical variables, the Chi-square test was applied, considering a significance level of 5%.

### Ethical aspects

The study was approved by the Institutional Ethics Committee (letter No. 255-GRPR-ESSALUD-2025) and was carried out in accordance with the principles of the Declaration of Helsinki. Informed consent was obtained from all participants and confidentiality was guaranteed through the anonymization of personal data. The database was stored in a protected file, with access restricted exclusively to the researchers.

## RESULTS

250 participants met the selection criteria, of whom 56.5% were male. In relation to age, 55.6% were between 61 and 80 years old, and 86.4% corresponded to adults over 60 years old. Regarding educational level, 66.4% had completed secondary education and 23.2% had higher studies. As for marital status, 76.4% of the participants had a stable partner (Table 1).

**Table 1:** Sociodemographic characteristics of patients under antihypertensive treatment at a tertiary-level hospital in Lima, 2025.

Characteristics		n	%
Sex			
	Male	141	56.4
	Female	109	43.6
Age			
	31−60 years	34	13.6
	61−80 years	139	55.6
	81‒100 years	77	30.8
Educational level			
	Primary	26	10.4
	Secondary	166	66.4
	Higher education	58	23.2
Marital status			
	Without partner	59	23.6
	With partner	191	76.4
	Total	250	100.0

All participants had at least one comorbidity with a clear predominance of chronic non-communicable diseases (79.8%), with a lower frequency of infectious diseases (1.3%) and neoplasms (3.4%). More than 60% had HTN for more than five years. The main reason for hospital admission was chronic non-communicable diseases (50.8%), followed by infectious diseases (27.6%), neoplasms (10%), and other conditions (11.6%); the most frequent specific causes being decompensations of cardiovascular diseases, respiratory infections, urinary tract infections, and complications associated with cancer.

42% of the participants presented low adherence to antihypertensive treatment, while 58% showed medium adherence, with no cases of high adherence recorded. In relation to the therapeutic regimen used, it was observed that monotherapy was the most used modality (48%). The most prescribed drug was losartan (34.8%), followed by nifedipine (16.8%) and enalapril (15.2%) (Table 2). The most frequent drug associations were ACEI + calcium channel blocker, ARB II + calcium channel blocker, and ARB II + calcium channel blocker + diuretic. All the drugs used were individual; there were no combination presentations in the institution's pharmacological formulary.

**Table 2:** Types of medications used by patients undergoing antihypertensive treatment at a tertiary-level hospital in Lima, 2025.

Medication		Frecuency	Percentage
Specific medication			
	Losartan	87	34.8
	Nifedipine	42	16.8
	Enalapril	38	15.2
	Irbesartan	37	14.8
	Valsartan	18	7.2
	Captopril	11	4.4
	Amlodipine	9	3.6
	Bisoprolol	7	2.8
	Spironolactone	1	0.4
Number of medications			
	Monotherapy	120	48.0
	2 medications	99	39.6
	3 or more medications	31	12.4
Total		250	100.0

Statistically significant differences were observed in pharmacological adherence according to educational level (p = 0.005) and the number of drugs (p < 0.001) (Table 3). In contrast, no significant differences were evidenced in adherence according to age (p = 0.658), sex (p = 0.566), or marital status (p = 0.570).

**Table 3:** Differences in pharmacological adherence to antihypertensive treatment in patients at a tertiary hospital in Lima, 2025.

Variable		Low Adherence (n = 105)	Medium adherence (n = 145)	p-value
		n (%)	n (%)	
Age				0.658
	31-60 years	15 (14.3)	19 (13.1)	
	61-80 years	58 (55.2)	81 (55.9)	
	81-100 years	32 (30.5)	45 (31.0)	
Sex				0.566
	Male	60 (57.1)	81 (55.9)	
	Female	45 (42.9)	64 (44.1)	
Educational level				0.005
	Primary	18 (17.1)	8 (5.5)	
	Secondary	70 (66.7)	96 (66.2)	
	Higher	17 (16.2)	41 (28.3)	
Marital status				0.570
	With partner	102 (78.5)	89 (74.2)	
	Without partner	28 (21.5)	31 (25.8)	
Number of drugs				<0.001
	1	53 (50.5)	67 (46.2)	
	2	37 (35.2)	50 (34.5)	
	3 or more	15 (14.3)	28 (19.3)	
Comorbidities				0.292
	1 to 2	60 (57.1)	91 (62. 8)	
	3 o more	45 (42.9)	54 (37.2)	
Time with HTN				0.717
	< 5 years	35 (33.3)	45 (31.0)	
	6-10 years	38 (36.2)	49 (33.8)	
	> 10 years	32 (30.5)	51 (35.2)	
Reason for admission				0.424
	NCDs	56 (53.3)	71 (49.0)	
	Infectious	27(25.7)	42 (29.0)	
	Neoplastic	10 (9.5)	15 (10.3)	
	Others	12 (11.5)	17 (11.7)	

NCDs: non-communicable chronic diseases

## DISCUSSION

The present study evidenced a high frequency of suboptimal pharmacological adherence to antihypertensive treatment in hospitalized patients at the tertiary level, a finding that is generally consistent with national and regional literature ^11^^,^^13^^,^^14^. It is noteworthy that no participant reached a high level of adherence, with a predominance of medium adherence (58%) and differences observed according to educational level and the number of drugs. This reinforces the need for multidisciplinary interventions, as proposed by the World Health Organization (WHO) in its framework for a comprehensive approach to therapeutic adherence ^1^^,^^4^.

The current classification of therapeutic adherence, based on the MMAS-8 scale, allows for greater sensitivity in identifying different levels of compliance compared to previous studies that used the MMAS-4 with a binary classification (adherent/non-adherent). In the national context, antecedents usually report "adequate" adherence rates without further stratification ^12^, while Pocohuanca-Ancco et al. (2021) evidenced a high proportion of non-adherence in a national hospital in Lima ^13^. In this context, our findings provide a more detailed and potentially more concerning view of the phenomenon, particularly in high-complexity hospital settings.

In the present study, males, older adults, secondary educational level, and married marital status predominated. The high proportion of patients with a stable partner could have limited the detection of significant differences in this variable, as has been reported in other studies ^15^.

Educational level showed significant differences with pharmacological adherence. This finding agrees with previous studies and with what was pointed out by the WHO, which recognizes the level of education as a key determinant for understanding treatment and therapeutic compliance. Nonetheless, the overall adherence identified in this study remains suboptimal, which coincides with global figures (42% low adherence) ^4^.

The low level of adherence observed is consistent with findings reported in Peru in 2021, where non-adherence rates between 70% and 75% are described in outpatients, which could be explained by differences in the clinical setting and the complexity of the patients ^11^^,^^16^. Although these studies used a different instrument (MMAS-4), the use of the MMAS-8 in the present study provides greater methodological rigor, given that it has demonstrated higher sensitivity compared to other questionnaires ^12^.

The clinical analysis evidenced a high prevalence of chronic non-communicable comorbidities (79.8%), in contrast to what was reported by Mendoza, who identified 25% in hypertensive patients treated in Lima. This difference could be explained by the clinical profile of patients hospitalized in a referral facility ^16^. This finding supports the evidence that the presence of multiple comorbidities complicates adherence and the comprehensive management of hypertension ^17^. Likewise, Altamirano et al. also reported a high frequency of non-communicable diseases, with diabetes being the most frequent comorbidity ^18^.

Monotherapy was the most used therapeutic regimen (48%), highlighting the use of losartan and nifedipine. Although this practice is aligned with international recommendations on the use of angiotensin II receptor antagonists (ARBs), a reduced use of thiazide diuretics was observed, despite being recommended as first-line by national ^19^ and international ^20^ guidelines. This difference may be due to clinical considerations such as the advanced age of the patients or the presence of comorbidities.

Significant differences were observed between the level of adherence and the number of antihypertensive medications. This finding agrees with that reported by Zapattini and Ortiz, who evidenced greater adherence in patients under combination therapy ^17^. These results suggest the need to re-evaluate the efficacy of current therapeutic regimens, especially in contexts of suboptimal blood pressure control.

The use of individual medications in the treatment of arterial hypertension is clinically relevant, as the prescription of multiple tablets in several daily doses constitutes a recognized factor for low therapeutic adherence. In this context, the absence of combination presentations of first-line antihypertensives in the social security pharmacological formulary represents an aspect that should be reviewed in order to propose changes that favor better adherence ^17^. Likewise, the low frequency of diuretic use is striking, in contrast to what is recommended by clinical practice guidelines ^19^^,^^20^. This could be explained by the greater clinical complexity of the evaluated patients, as well as by the renal involvement associated with the prolonged progression of hypertension and the presence of comorbidities.

The findings presented suggest directing interventions toward improving therapeutic adherence, prioritizing differentiated educational strategies adapted to the patients' level of education. Multidisciplinary approaches should also be encouraged and continuous adherence monitoring systems established as an integral part of arterial hypertension management ^4^^,^^19^^,^^20^.

Among the limitations of the study is that only patients on pharmacological antihypertensive treatment were included, so the results are not extrapolable to people with a diagnosis of arterial hypertension who do not receive medication, which could overestimate the observed levels of adherence. Likewise, the types of medications or their combinations were not evaluated in detail, with the analysis being limited to the number of drugs used.

Additionally, its cross-sectional design prevents establishing causal relationships and, having been conducted in a single referral hospital center, the results are not representative of the primary and secondary levels of care. Nevertheless, the study provides relevant local evidence, using a validated instrument (MMAS-8) and a sample of adequate size. Future multicenter studies, with a longitudinal design and incorporating psychosocial and economic variables, will allow for a deeper understanding of the differences in therapeutic adherence and validate the findings obtained.

Finally, it is concluded that in hypertensive patients hospitalized at the tertiary level, medium and low pharmacological adherence predominate, with no cases of high adherence recorded. Differences in adherence were identified according to educational level and the number of drugs. These findings highlight the need to design educational strategies adapted to the sociocultural profile of the patients, as well as to consider the implementation of more effective therapeutic regimens. Furthermore, the importance of continuously monitoring adherence as a fundamental part of the comprehensive management of arterial hypertension is emphasized.
