# Supplementary figures and images for: Adherence to antihypertensive treatment in patients at a tertiary hospital in Lima, Peru
Source: Rev Peru Med Exp Salud Publica. 2026 Mar 27;43(1):98–103. doi: 10.17843/rpmesp.2026.431.15462 (PMC13245980; doi:10.17843/rpmesp.2026.431.15462)

**MATERIAL SUPLEMENTARIO**

Anexo 1

Escala de adherencia a la medicación de Morisky 8 ítems (MMAS-8)


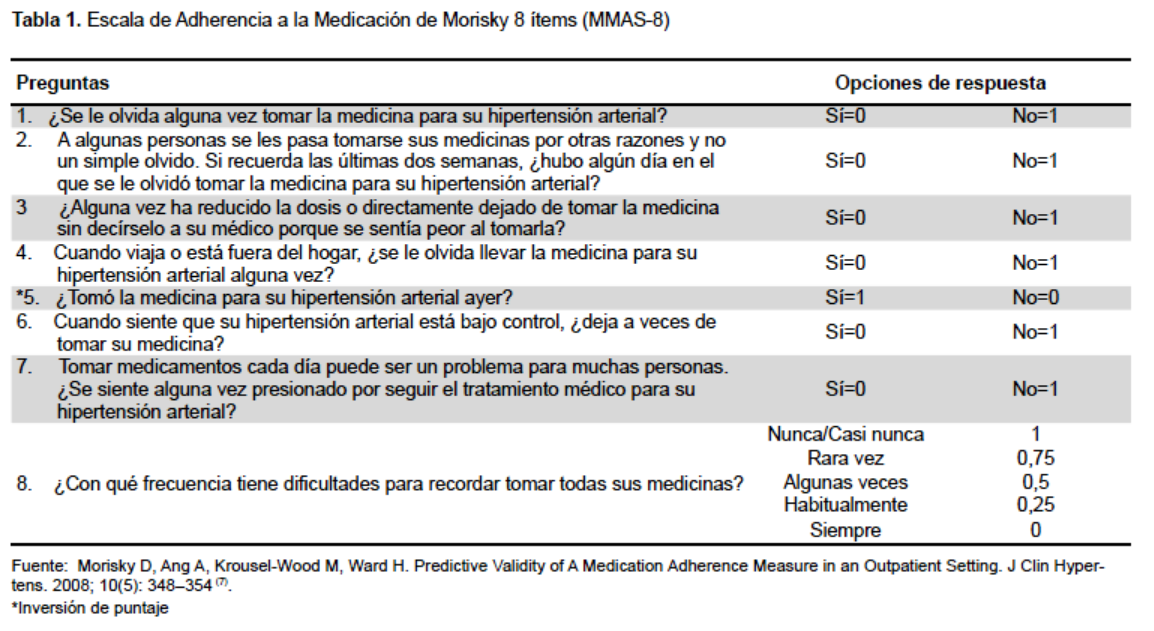

Supplement: Supplementary material. — Available in the electronic version of the RPMESP. [file rpmesp-43-01-15462-s001.docx]
