# Supplementary material for: Adherence to antihypertensive treatment in patients at a tertiary hospital in Lima, Peru
Source: Rev Peru Med Exp Salud Publica. 2026 Mar 27;43(1):98–103. doi: 10.17843/rpmesp.2026.431.15462 (PMC13245980; doi:10.17843/rpmesp.2026.431.15462)
Supplement: Translation

# Translation: Adherencia al tratamiento antihipertensivo en pacientes de un hospital terciario en Lima, Perú

**Authors:** Angy Aguilar-Lizarme, José Amado-Tineo

**Affiliations:** 1 Facultad de Medicina, Universidad Nacional Mayor de San Marcos, Lima, Perú.; 2 Hospital Nacional Edgardo Rebagliati Martins, Lima, Perú.

**Keywords:** Cumplimiento y Adherencia al Tratamiento, Hipertensión, Antihipertensivos

## Abstract

En Perú existen escasos reportes sobre la adherencia farmacológica en pacientes con hipertensión arterial. En este contexto, el presente estudio tuvo como objetivo determinar la adherencia al tratamiento antihipertensivo en pacientes atendidos en un hospital de nivel terciario de la seguridad social en Lima. Se realizó un estudio transversal en 250 pacientes hospitalizados, mayores de 18 años, con diagnóstico de hipertensión arterial primaria en tratamiento farmacológico. La adherencia se evaluó mediante el cuestionario de Morisky (MMAS-8), y se recolectaron variables como medicación indicada, edad, sexo, estado civil y nivel educativo. El 56,4% de los participantes fueron varones y el 86,4% tenía más de 60 años. Se observó que el 42% presentó baja adherencia farmacológica y el 58% adherencia media, sin registrarse casos de adherencia alta. La monoterapia fue el esquema más frecuente (48%), siendo losartán, nifedipino y enalapril los fármacos más prescritos. Finalmente, se identificaron diferencias significativas entre la adherencia al tratamiento antihipertensivo con el nivel educativo y el número de fármacos.

## INTRODUCCIÓN

La hipertensión arterial (HTA) es la condición patológica cardiovascular más común entre los adultos, afectando a más de la cuarta parte de la población mundial, lo que equivale a más de mil millones de personas ^1^. Se clasifica en HTA esencial o primaria, que representa aproximadamente el 90-95% de los casos y en hipertensión secundaria (5-10%), asociada a enfermedades renales, endocrinas, uso de medicamentos entre otras causas. La adherencia al tratamiento antihipertensivo constituye un determinante clave del control de la HTA. Diversos factores, como la complejidad del régimen terapéutico, la polifarmacia y variables sociodemográficas, pueden influir en el cumplimiento terapéutico ^2^^,^^3^.

A nivel global, se estima que el 43% de los pacientes hipertensos presentan una baja adherencia al tratamiento, con cifras más altas en países de ingresos bajos y medios ^4^. Como resultado, el control efectivo de la presión arterial es limitado, ya que solo el 13,8% de los casos estaban controlados en el 2020 ^5^. La evidencia ha demostrado que el incumplimiento terapéutico se asocia de manera directa con un peor control de la presión arterial, mayor progresión del daño de órgano blanco y un incremento significativo del riesgo cardiovascular ^6^.

Diversos estudios internacionales han identificado factores asociados con la adherencia, como la edad, nivel educativo, presencia de comorbilidades y complejidad del tratamiento ^6^. En Letonia, por ejemplo, se reportó una tasa de incumplimiento del 46,2%, con mayor adherencia entre adultos mayores ^7^. En Arabia Saudí, solo el 42,2% de los pacientes seguía adecuadamente el régimen de medicación, influenciados por enfermedades coexistentes y el uso de múltiples fármacos ^8^. En Brasil, el 38% de los pacientes sin educación secundaria presentaban mejor adherencia en comparación con el 12,5% de los terminaron secundaria ^9^.

En Perú, existen retos importantes para garantizar el control de la HTA, debido a limitaciones en el monitoreo del riesgo cardiovascular y la distribución adecuada de medicamentos ^10^. Se ha evidenciado que las tasas de adherencia al tratamiento oscilan entre el 30% y 41% en pacientes adultos atendidos en establecimientos del primer y segundo nivel de atención, lo que impacta negativamente en los resultados de salud y los costos del sistema sanitario ^11^. En este contexto, se busca determinar el nivel de adherencia farmacológica al tratamiento antihipertensivo e identificar diferencias según características de los pacientes en un hospital de referencia.

**MENSAJE CLAVE**

Motivación para realizar el estudio. La adherencia al tratamiento antihipertensivo es un aspecto poco evaluado en nuestro medio, pese a ser un componente clave para el adecuado control de la hipertensión arterial.

Principales hallazgos. El 58% de los pacientes hospitalizados en un establecimiento de nivel terciario presentó adherencia media, mientras que el 42% mostró baja adherencia. Se identificaron diferencias significativas en la adherencia según el nivel educativo y el número de fármacos consumidos.

Implicancias en salud pública. La identificación de diferencias en la adherencia al tratamiento antihipertensivo permite reconocer posibles puntos de intervención y orientar el diseño de estrategias para optimizar el control de la hipertensión arterial.

## EL ESTUDIO

### Diseño y población

Se realizó un estudio observacional de corte transversal. La población estuvo conformada por pacientes en tratamiento farmacológico antihipertensivo hospitalizados en los servicios de medicina interna del Hospital Nacional Edgardo Rebagliati Martins, entre febrero y marzo de 2025. Este establecimiento de nivel terciario cuenta con todas las especialidades médicas, dispone de aproximadamente 1600 camas hospitalarias y seis servicios de medicina interna, cada uno con un promedio de 40 camas.

### Selección, muestra y muestreo

El tamaño muestral se estimó en 227 participantes mediante el programa Epi Info versión 7.2.5, considerando una población de tamaño desconocido, un margen de error del 5%, un nivel de confianza del 95% y un efecto de diseño de 1. Se incluyó de manera consecutiva a pacientes mayores de 18 años, con diagnóstico de hipertensión arterial primaria y en tratamiento farmacológico antihipertensivo por más de tres meses, que otorgaron su consentimiento informado. Se excluyeron aquellos con evidencia de hipertensión secundaria, inestabilidad hemodinámica, datos incompletos o con limitaciones neurológicas o físicas que impidieran responder la encuesta.

### Variables

Mediante la revisión de la historia clínica hospitalaria se identificaron características sociodemográficas: edad (años registrados), sexo (masculino y femenino), nivel educativo (primaria, secundaria y superior), estado civil (con o sin pareja) y variables clínicas (comorbilidad, motivo de ingreso y tiempo de HTA) de los participantes.

Se evaluó la adherencia al tratamiento farmacológico antihipertensivo previo a la hospitalización utilizando el cuestionario MMAS-8 (8-item Morisky Medication Adherence Scale) ^12^ y se registró el tipo y número de fármacos utilizados. Los datos fueron recolectados por un egresado de medicina mediante entrevista directa al paciente, no aceptando respuestas de familiares ni acompañantes.

El MMAS-8 consta de siete ítems con respuesta dicotómica (sí = 0 puntos / no = 1 punto), mientras que el octavo ítem presenta una escala de respuesta de cinco puntos tipo Likert. La suma de puntaje clasifica en: baja adherencia (menos de 6), adherencia media (6-7) y alta adherencia (8 o más) (material suplementario).

### Análisis estadístico

Los datos fueron digitados en Microsoft Excel 2017 y posteriormente analizados con el programa estadístico SPSS versión 25. Las variables se categorizaron y se describieron mediante frecuencias absolutas y porcentajes. Para evaluar diferencias entre las variables categóricas, se aplicó la prueba de Chi-cuadrado, considerando un nivel de significancia del 5%.

### Aspectos éticos

El estudio fue aprobado por el Comité de Ética Institucional (carta N.° 255-GRPR-ESSALUD-2025) y se llevó a cabo conforme a los principios de la Declaración de Helsinki. Se obtuvo el consentimiento informado de todos los participantes y se garantizó la confidencialidad mediante la anonimización de los datos personales. La base de datos fue almacenada en un archivo protegido, con acceso restringido exclusivamente a los investigadores.

## RESULTADOS

Cumplieron los criterios de selección 250 participantes, de los cuales el 56,5% fueron de sexo masculino. En relación con la edad, el 55,6% tenía entre 61 y 80 años, y el 86,4% correspondía a adultos mayores de 60 años. Respecto al nivel educativo, el 66,4% había completado la educación secundaria y el 23,2% contaba con estudios superiores. En cuanto al estado civil, el 76,4% de los participantes tenía una pareja estable (tabla 1).

**Tabla 1:** Características sociodemográficas de los pacientes en tratamiento antihipertensivo de un hospital del nivel terciario de Lima, 2025.

Características		n	%
Sexo			
	Masculino	141	56,4
	Femenino	109	43,6
Edad			
	31−60 años	34	13,6
	61−80 años	139	55,6
	81‒100 años	77	30,8
Nivel educativo			
	Primaria	26	10,4
	Secundaria	166	66,4
	Superior	58	23,2
Estado civil			
	Sin pareja	59	23,6
	Con pareja	191	76,4
	Total	250	100,0

Todos los participantes tenían al menos una comorbilidad con claro predominio de patologías crónicas no transmisibles (79,8%), con menor frecuencia de enfermedades infecciosas (1,3%) y neoplasias (3,4%). Más del 60% presentaban HTA por más de cinco años. El principal motivo de ingreso a hospitalización fueron las enfermedades crónicas no transmisibles (50,8%), seguidas de enfermedades infecciosas (27,6%), neoplasias (10%) y otras condiciones (11,6%); siendo las causas específicas más frecuentes las descompensaciones de enfermedades cardiovasculares, infecciones respiratorias, infecciones urinarias y complicaciones asociadas a cáncer.

El 42% de los participantes presentó una baja adherencia al tratamiento antihipertensivo, mientras que el 58% evidenció una adherencia media, sin registrarse caso de alta adherencia. En relación con el esquema terapéutico empleado, se observó que la monoterapia fue la modalidad más utilizada (48%). El fármaco más prescrito fue losartán (34,8%), seguido por nifedipino (16,8%) y enalapril (15,2%) (tabla 2). La asociación de medicamentos más frecuentes fueron IECA + calcioantagonista, ARA II + calcioantagonista y ARA II + calcioantagonista + diurético. Todos los fármacos utilizados eran individuales, no había presentaciones combinadas en el petitorio farmacológico de la institución.

**Tabla 2:** Tipo de fármaco que emplean los pacientes en tratamiento antihipertensivo de un hospital del nivel terciario de Lima, 2025.

Fármaco		n	%
Fármaco específico			
	Losartán	87	34,8
	Nifedipino	42	16,8
	Enalapril	38	15,2
	Irbesartán	37	14,8
	Valsartán	18	7,2
	Captopril	11	4,4
	Amlodipino	9	3,6
	Bisoprolol	7	2,8
	Espironolactona	1	0,4
Número de fármacos			
	Monoterapia	120	48,0
	2 fármacos	99	39,6
	3 o más fármacos	31	12,4
Total		250	100,0

Se observaron diferencias estadísticamente significativas en la adherencia farmacológica según el nivel educativo (p = 0,005) y el número de fármacos (p < 0,001) (tabla 3). En contraste, no se evidenciaron diferencias significativas en la adherencia según la edad (p = 0,658), el sexo (p = 0,566) ni el estado civil (p = 0,570).

**Tabla 3:** Diferencias en la adherencia farmacológica al tratamiento antihipertensivo en pacientes de un hospital terciario en Lima, 2025.

Variable		Adherencia Baja (n = 105)	Adherencia media (n = 145)	Valor de p
		n (%)	n (%)	
Edad				0,658
	31-60 años	15 (14,3)	19 (13,1)	
	61-80 años	58 (55,2)	81 (55,9)	
	81-100 años	32 (30,5)	45 (31,0)	
Sexo				0,566
	Masculino	60 (57,1)	81 (55,9)	
	Femenino	45 (42,9)	64 (44,1)	
Nivel educativo				0,005
	Primaria	18 (17,1)	8 (5,5)	
	Secundaria	70 (66,7)	96 (66,2)	
	Superior	17 (16,2)	41 (28,3)	
Estado Civil				0,570
	Con pareja	102 (78,5)	89 (74,2)	
	Sin pareja	28 (21,5)	31 (25,8)	
Número de fármacos				<0,001
	1	53 (50,5)	67 (46,2)	
	2	37 (35,2)	50 (34,5)	
	3 o más	15 (14,3)	28 (19,3)	
Comorbilidades				0,292
	1 a 2	60 (57,1)	91 (62, 8)	
	3 o más	45 (42,9)	54 (37,2)	
Tiempo de HTA				0,717
	< 5 años	35 (33,3)	45 (31,0)	
	6-10 años	38 (36,2)	49 (33,8)	
	> 10 años	32 (30,5)	51 (35,2)	
Motivo de ingreso				0,424
	ECNT	56 (53,3)	71 (49,0)	
	Infecciosas	27(25,7)	42 (29,0)	
	Neoplásicas	10 (9,5)	15 (10,3)	
	Otros	12 (11,5)	17 (11,7)	

ECNT: enfermedades crónicas no transmisibles

## DISCUSIÓN

El presente estudio evidenció una elevada frecuencia de adherencia farmacológica subóptima al tratamiento antihipertensivo en pacientes hospitalizados en el nivel terciario, hallazgo que guarda coherencia general con la literatura nacional y regional ^11^^,^^13^^,^^14^. Llama la atención que ningún participante alcanzó un nivel alto de adherencia, observándose predominio de adherencia media (58%) con diferencias según nivel educativo y el número de fármacos. Esto refuerza la necesidad de intervenciones multidisciplinarias, como lo propone la Organización Mundial de la Salud (OMS) en su marco de abordaje integral de la adherencia terapéutica ^1^^,^^4^.

La clasificación actual de la adherencia terapéutica, basada en la escala MMAS-8, permite una mayor sensibilidad para identificar distintos niveles de cumplimiento, en comparación con estudios previos que emplearon la MMAS-4 con una clasificación binaria (adherente/no adherente). En el ámbito nacional, los antecedentes suelen reportar tasas de adherencia «adecuada» sin mayor estratificación ^12^, mientras que Pocohuanca-Ancco *et al*. (2021) evidenciaron una alta proporción de no adherencia en un hospital nacional de Lima ^13^. En este contexto, nuestros hallazgos aportan una visión más detallada y, potencialmente, más preocupante del fenómeno, particularmente en entornos hospitalarios de mayor complejidad.

En el presente estudio predominó el sexo masculino, los adultos mayores, el nivel educativo secundario y el estado civil casado. La alta proporción de pacientes con pareja estable podría haber limitado la detección de diferencias significativas en esta variable, como se ha reportado en otros estudios ^15^.

El nivel educativo presentó diferencias significativas con la adherencia farmacológica. Este hallazgo concuerda con estudios previos y con lo señalado por la OMS, que reconoce al nivel de instrucción como un determinante clave para la comprensión del tratamiento y el cumplimiento terapéutico. No obstante, la adherencia general identificada en este estudio sigue siendo subóptima, lo cual coincide con las cifras a nivel global (42% de baja adherencia) ^4^.

El bajo nivel de adherencia observado es consistente con hallazgos reportados en Perú en 2021, donde se describen tasas de no adherencia entre el 70% y 75% en pacientes ambulatorios, lo que podría explicarse por diferencias en el entorno clínico y la complejidad de los pacientes ^11^^,^^16^. Si bien estos estudios emplearon un instrumento distinto (MMAS-4), el uso del MMAS-8 en el presente estudio aporta mayor rigor metodológico, dado que ha demostrado una mayor sensibilidad en comparación con otros cuestionarios ^12^.

El análisis clínico evidenció una alta prevalencia de comorbilidades crónicas no transmisibles (79,8%), en contraste con lo reportado por Mendoza, quien identificó un 25% en pacientes hipertensos atendidos en Lima. Esta diferencia podría explicarse por el perfil clínico de los pacientes hospitalizados en un establecimiento de referencia ^16^. Este hallazgo respalda la evidencia de que la presencia de múltiples comorbilidades dificulta la adherencia y el manejo integral de la hipertensión ^17^. Asimismo, Altamirano *et al*. también reportaron una alta frecuencia de enfermedades no transmisibles, siendo la diabetes la comorbilidad más frecuente ^18^.

La monoterapia fue el esquema terapéutico más empleado (48%), destacando el uso de losartán y nifedipino. Aunque esta práctica está alineada con las recomendaciones internacionales sobre el uso de antagonistas de los receptores de angiotensina II (ARA-II), se observó un uso reducido de diuréticos tiazídicos, pese a ser recomendados como primera línea por las guías nacionales ^19^ e internacionales ^20^. Esta diferencia puede deberse a consideraciones clínicas como la edad avanzada de los pacientes o la presencia de comorbilidades.

Se observaron diferencias significativas entre el nivel de adherencia y el número de medicamentos antihipertensivos. Este hallazgo concuerda con lo reportado por Zapattini y Ortiz, quienes evidenciaron una mayor adherencia en pacientes bajo terapia combinada ^17^. Estos resultados sugieren la necesidad de reevaluar la eficacia de los esquemas terapéuticos actuales, especialmente en contextos de control subóptimo de la presión arterial.

El uso de medicamentos individuales en el tratamiento de la hipertensión arterial es clínicamente relevante, ya que la prescripción de múltiples comprimidos en varias tomas diarias constituye un factor reconocido de baja adherencia terapéutica. En este contexto, la ausencia de presentaciones combinadas de antihipertensivos de primera línea en el petitorio farmacológico de la seguridad social representa un aspecto que debería revisarse, con el fin de proponer cambios que favorezcan una mejor adherencia ^17^. Asimismo, resulta llamativa la baja frecuencia de uso de diuréticos, en contraste con lo recomendado por las guías de práctica clínica ^19^^,^^20^. Esto podría explicarse por la mayor complejidad clínica de los pacientes evaluados, así como por el compromiso renal asociado a la evolución prolongada de la hipertensión y la presencia de comorbilidades.

Los hallazgos presentados sugieren orientar intervenciones a mejorar la adherencia terapéutica, priorizando estrategias educativas diferenciadas, adaptadas al nivel de instrucción de los pacientes. También se debe fomentar el abordaje multidisciplinario y establecer sistemas de seguimiento continuo de la adherencia como parte integral del manejo de la hipertensión arterial ^4^^,^^19^^,^^20^.

Entre las limitaciones del estudio, se encuentra que solo se incluyó a pacientes en tratamiento farmacológico antihipertensivo, por lo que los resultados no son extrapolables a personas con diagnóstico de hipertensión arterial que no reciben medicación, lo que podría sobreestimar los niveles de adherencia observados. Asimismo, no se evaluaron en detalle los tipos de medicamentos ni sus combinaciones, limitándose el análisis al número de fármacos utilizados.

Adicionalmente, su diseño transversal impide establecer relaciones de causalidad y, al haberse realizado en un único centro hospitalario de referencia, los resultados no son representativos de los niveles primario y secundario de atención. No obstante, el estudio aporta evidencia local relevante, utilizando un instrumento validado (MMAS-8) y una muestra de tamaño adecuado. Futuros estudios multicéntricos, de diseño longitudinal e incorporando variables psicosociales y económicas, permitirán profundizar en la comprensión de las diferencias en la adherencia terapéutica y validar los hallazgos obtenidos.

Finalmente, se concluye que en pacientes hipertensos hospitalizados en el nivel terciario predomina una adherencia farmacológica media y baja, sin registrarse casos de adherencia alta. Se identificaron diferencias en la adherencia según el nivel educativo y el número de fármacos. Estos hallazgos evidencian la necesidad de diseñar estrategias educativas adaptadas al perfil sociocultural de los pacientes, así como de considerar la implementación de esquemas terapéuticos más efectivos. Asimismo, se resalta la importancia de monitorear de manera continua la adherencia como parte fundamental del abordaje integral de la hipertensión arterial.
